# Anastomotic leakage after esophagectomy possibly caused by compression of the gastric conduit behind the sternoclavicular joint: a report of three cases

**DOI:** 10.1186/s40792-021-01250-3

**Published:** 2021-07-13

**Authors:** Yasunori Kurahashi, Yudai Hojo, Tatsuro Nakamura, Tsutomu Kumamoto, Yoshinori Ishida, Hisashi Shinohara

**Affiliations:** Department of Gastroenterological Surgery, Hyogo College of Medicine, 1-1 Mukogawa-cho, Nishinomiya, Hyogo 663-8501 Japan

**Keywords:** Esophagectomy, Anastomotic leakage, Sternoclavicular joint, Compression, Indocyanine green fluorescence imaging

## Abstract

**Background:**

The narrowness of the thoracic inlet is often a problem in retrosternal reconstruction after esophagectomy. We report here three cases in which compression of the gastric conduit behind the sternoclavicular joint possibly caused anastomotic leakage.

**Case presentations:**

The first case was a 71-year-old man who underwent subtotal esophagectomy for upper esophageal cancer followed by retrosternal reconstruction. On postoperative day 2, he developed septic shock and underwent reoperation because of a necrotic gastric conduit. The tip of the conduit above the manubrium was necrotic due to strangulation as a result of compression by the sternoclavicular joint. The second and third cases were a 50-year-old woman and a 71-year-old man who underwent subtotal esophagectomy for middle and lower esophageal cancer, respectively, followed by retrosternal reconstruction. Despite indocyanine green fluorescence imaging indicating adequate blood flow in both cases, the tip of the conduit appeared pale and congested because of compression by the sternoclavicular joint after anastomosis. Postoperatively, these two patients developed anastomotic leakage that was confirmed endoscopically on the ventral side of the gastric wall that had been pale intraoperatively.

**Conclusions:**

When performing reconstruction using the retrosternal route after esophagectomy, it is important to ensure that compression by the sternoclavicular joint does not have an adverse impact on blood flow at the tip of the gastric conduit.

## Introduction

Three reconstruction routes possible after esophagectomy are posterior mediastinal, retrosternal and percutaneous. Although the retrosternal route is often chosen for safety in the event of postoperative complications, it has a drawback in terms of higher incidence of anastomotic leakage and stenosis [1–4]. One of the reasons for this problem is that the reconstructed organ which passes through the narrow thoracic inlet with abrupt flexion around the trachea is compressed by the sternoclavicular joint (SCJ). We recently reported that anastomosis behind the SCJ was associated with increased incidence of postoperative stenosis [1]. To avoid this problem, the anastomosis should be located away from the SCJ. However, if the anastomotic site is placed above the manubrium, protrusion of the SCJ may compress the tip of the gastric conduit and cause a reduction in blood flow. Here, we report three cases in which compression by the SCJ caused impaired gastric blood flow resulting in anastomotic leakage.

## Case presentations

### Case 1

The patient was a 71-year-old man who had a history of chronic obstructive pulmonary disease, right upper lobectomy for lung cancer and partial liver resection for hepatocellular carcinoma. He underwent subtotal esophagectomy for upper esophageal cancer with 3-field lymph node dissection via video-assisted thoracic surgery (VATS), followed by retrosternal reconstruction. The 4.0-cm-wide gastric conduit was created extracorporeally and pulled up to the neck via the retrosternal route. The remnant cervical esophagus was fully mobilized and transected near skin level. An end-to-end hand-sewn anastomosis was fashioned and placed above the manubrium by pulling the conduit down from the abdominal cavity to straighten the digestive tract.

**Fig. 1:**
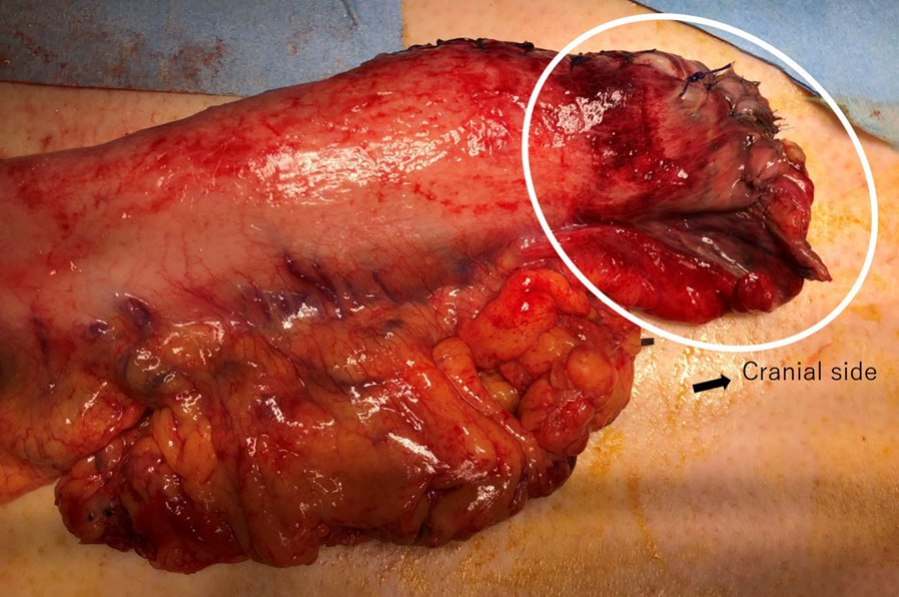
Intraoperative findings in case 1 at the time of reoperation. The tip of the gastric conduit above the manubrium was necrotic from strangulation as a result of compression by the sternoclavicular joint (white circle)

On postoperative day (POD) 2, he developed septic shock and underwent reoperation after emergency endoscopy revealed necrosis of the conduit. During the second operation, the tip of the conduit above the manubrium was found to be necrotic from strangulation as a result of compression by the SCJ (Fig. 1). The necrotic conduit was removed, and an esophageal fistula was constructed in the left side of the neck for a second reconstruction to be performed later.

### Case 2

**Fig. 2:**
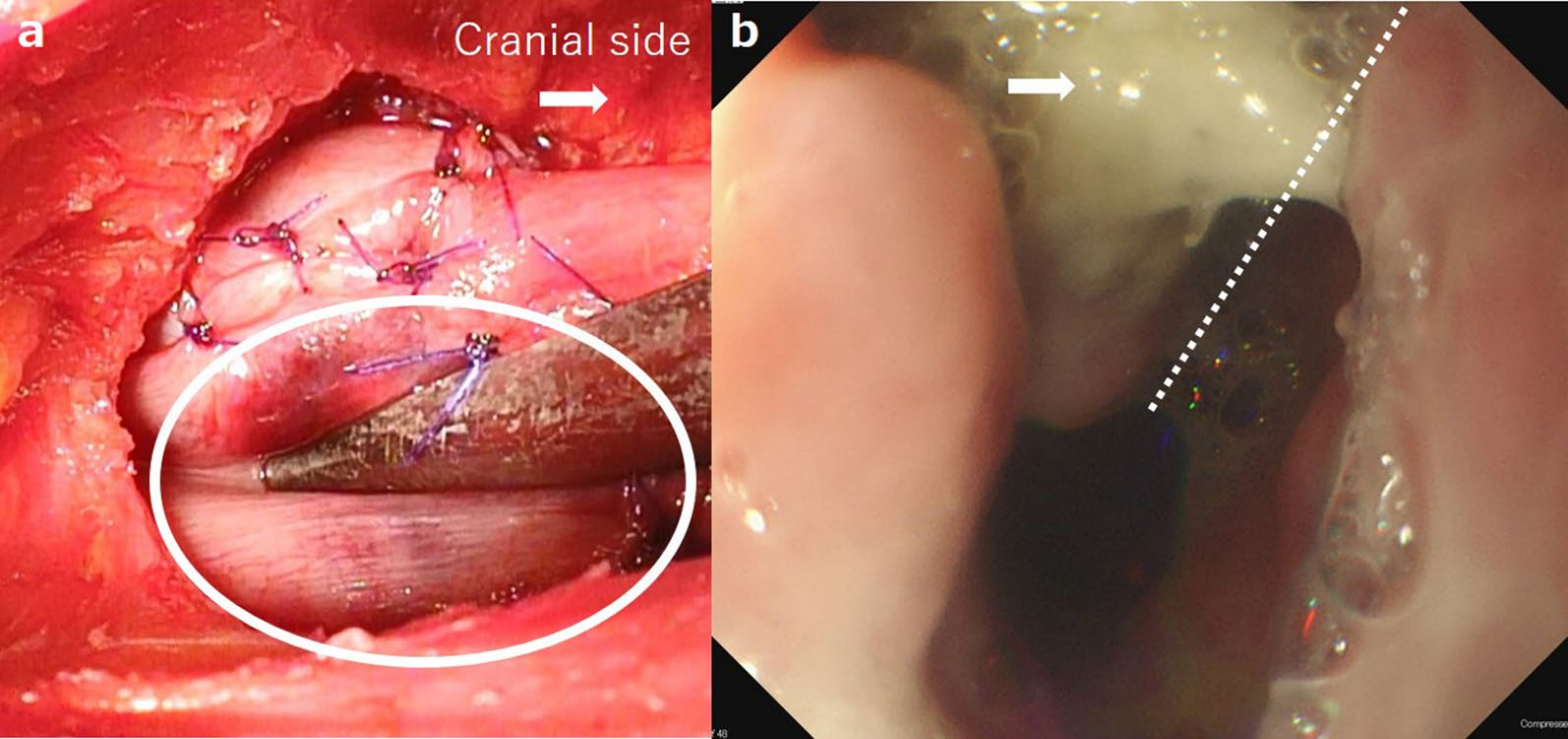
**a** Anastomosis at the end of surgery in case 2. The tip of the gastric conduit appeared pale with the trace of the upper end of the manubrium as the clear boundary (white circle). **b** Endoscopic findings in case 2 on postoperative day 8. The dashed line indicates the staple line of the conduit at the lesser curvature. Leakage was confirmed at the ventral side of the tip of the conduit (white arrow)

In this case, a 50-year-old woman underwent two cycles of neoadjuvant chemotherapy (fluorouracil and cisplatin) and subtotal esophagectomy for middle esophageal cancer with 3-field lymph node dissection via VATS, followed by retrosternal reconstruction. The gastric conduit was 4.0 cm wide, and the reconstruction procedure was the same as in case 1. The anastomotic site was determined by indocyanine green (ICG) fluorescence imaging before pulling up. After the anastomosis, the tip of the conduit appeared pale with the trace of the upper end of the manubrium as the clear boundary (Fig. 2a). Although re-anastomosis was considered, it was not performed because ICG fluorescence imaging had indicated adequate blood flow at the anastomotic site.

She developed anastomotic leakage on POD 8, which was confirmed endoscopically on the ventral side of the tip of the gastric conduit, which had been pale intraoperatively (Fig. 2b). We determined the cause of the anastomotic leakage to be circulatory disturbance at the tip of the conduit behind the SCJ. She recovered with cervical drainage, but developed anastomotic stenosis afterwards.

### Case 3

The patient was a 71-year-old man who underwent three cycles of neoadjuvant chemotherapy (fluorouracil, cisplatin and docetaxel) and subtotal esophagectomy for lower esophageal cancer with 2-field lymph node dissection via VATS, followed by retrosternal reconstruction with a 3.5-cm-wide gastric conduit. Although sufficient blood flow to the anastomotic site had been confirmed by ICG fluorescence imaging, the tip of the conduit looked pale after anastomosis. Anastomotic leakage developed on POD 5. Endoscopic findings indicated that the cause of leakage was circulatory disturbance at the tip of the conduit behind the SCJ, as in case 2. He recovered with cervical drainage, but also subsequently developed anastomotic stenosis.

## Discussion

One of the drawbacks of the retrosternal route after esophagectomy is the narrowness of the thoracic inlet where the SCJ is located and backward protrusion of the manubrium and the clavicular head, which compress the gastrointestinal tract after reconstruction, resulting in postoperative problems. We recently reported that anastomosis behind the SCJ is associated with an increased incidence of anastomotic stenosis. Considering that the rate of stenosis in the anastomosis above the manubrium was as low as 4.3% in that study, we pulled up the gastric conduit sufficiently so that the anastomosis was not behind the SCJ. However, in a subsequent series, we encountered three cases of leakage that were probably due to compression by the SCJ. Placing the anastomosis above the SCJ also poses certain risks.

The main problem with compression by the SCJ is impaired blood flow at the tip of the gastric conduit. When the compression is severe, the blood flow is blocked and the gastric wall becomes ischemic. In case 1, judging from the specimen taken at the time of reoperation on POD 2, the tip of the conduit had become strangled at the thoracic inlet, resulting in necrosis due to ischemia. However, when the compression is mild, congestion rather than ischemia may cause circulatory disturbance, resulting in mucosal damage, ulceration or poor healing, followed by anastomotic leakage. We speculate that this happened in cases 2 and 3 because the time to onset of leakage was relatively long.

**Fig. 3:**
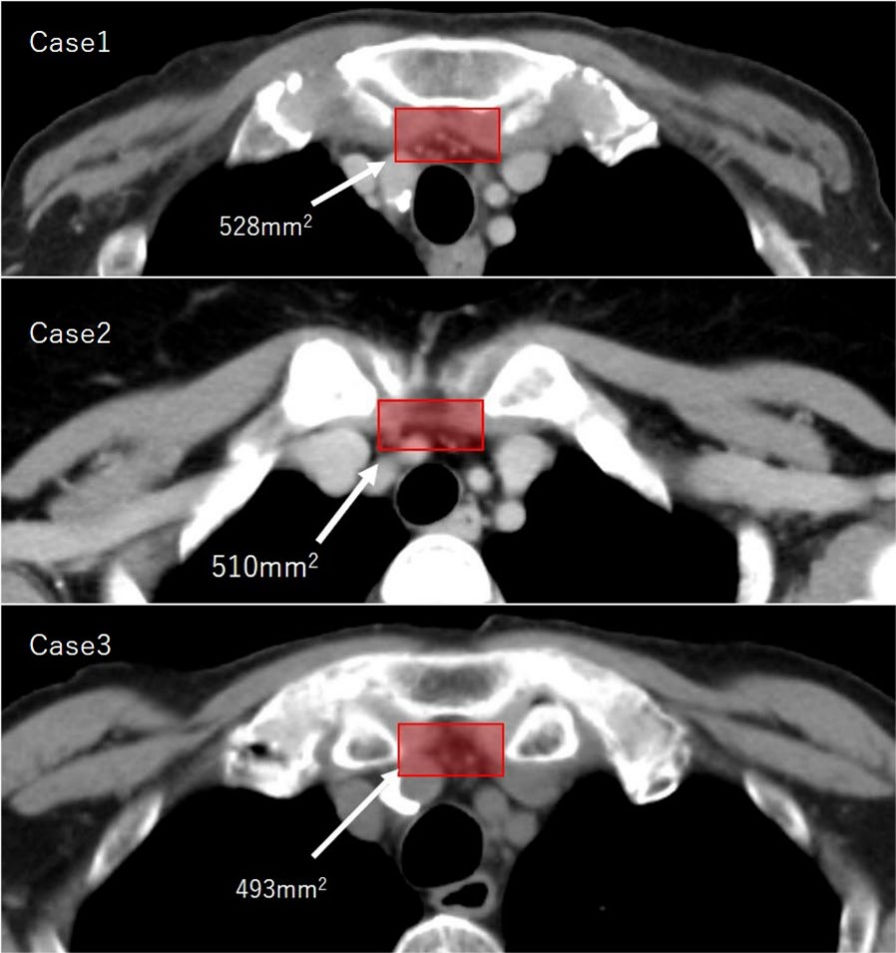
Measurement of the thoracic inlet space (TIS) in each case. The TIS was defined as the inter-clavicular distance multiplied by the sterno-brachiocephalic distance in the axial section of the cervical chest on preoperative computed tomography scans. In all the cases, the TIS was < 700 mm^2^

**Fig. 4:**
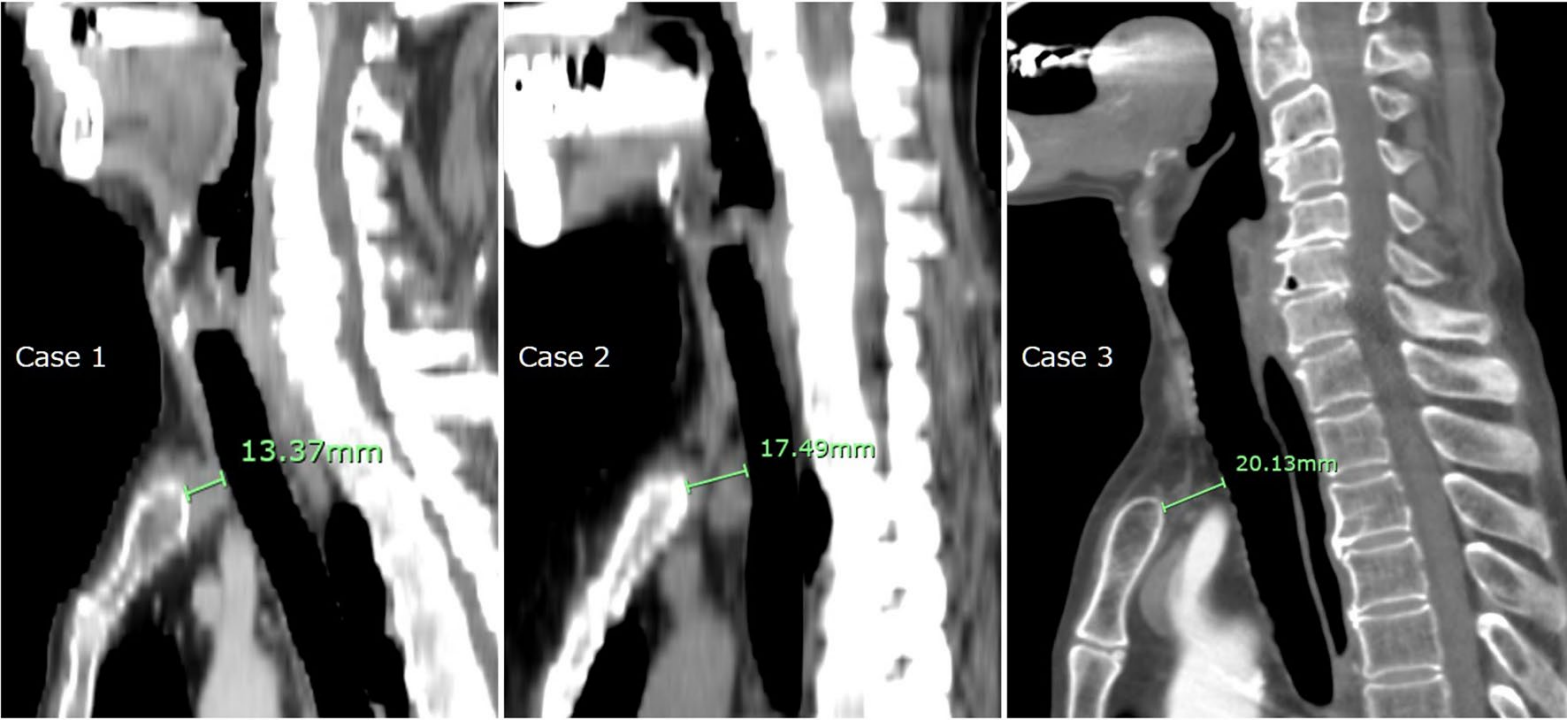
Measurement of the sterno-tracheal distance (STD) in each case. The STD was defined as the minimum distance from the sternum to the anterior wall of the trachea in the sagittal section of cervical chest on preoperative computed tomography scans. None of the cases had an STD < 13 mm

Preoperative evaluation of the narrowness of the inlet space is important to reduce the complications arising from compression by the SCJ. Several studies have evaluated the size of the thoracic inlet space [2–4]. Kunisaki et al. recommended a thoracic inlet space (TIS) of > 700 mm^2^ and resection of bony structures to avoid anastomotic leakage when the space is narrow [2]. Inoue et al. concluded that the sterno-tracheal distance (STD) was an independent risk factor for anastomotic leakage in the retrosternal route and that this route should be avoided in patients with an STD < 13 mm [3]. In all three cases in the present study, the TIS was < 700 mm^2^, meaning that the inlet space was narrower than that recommended by Kunisaki et al. However, the STD was > 13 mm in all three cases, meaning that the STD was of sufficient width to perform retrosternal reconstruction (Figs. 3, 4). Therefore, we considered that it would be difficult to evaluate the narrowness of the inlet space on preoperative computed tomography scans.

**Fig. 5:**
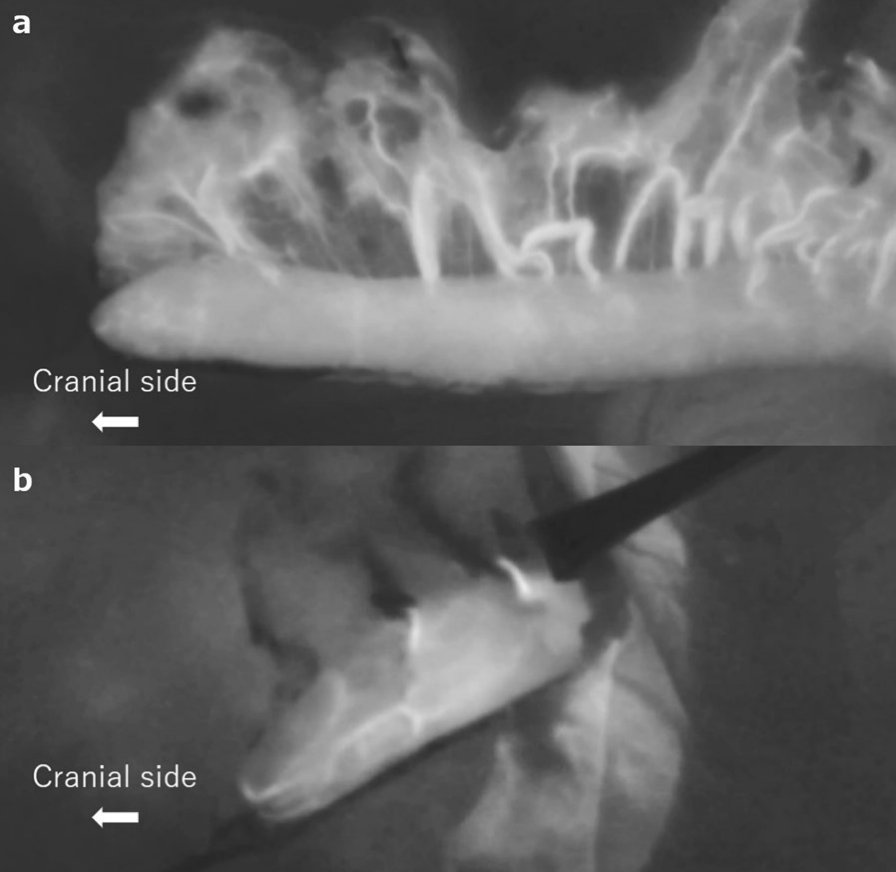
Indocyanine green fluorescence imaging in another recent case. We adopted a method of confirming the blood flow in the conduit both before (**a**) and after (**b**) pulling up

ICG fluorescence imaging is often used to evaluate the blood flow in the gastric wall for safe anastomosis [5, 6]. Koyanagi et al. reported that intraoperative evaluation of the speed of blood flow by ICG fluorescence is a useful means to predict the risk of anastomotic leakage, and it is recommended to use the gastric wall for anastomosis where blood flow is confirmed within a certain period of time [5]. We also performed ICG fluorescence imaging and confirmed good blood flow in the gastric wall but could not avoid anastomotic leakage. The reason was thought to be that the compression at the thoracic inlet after pulling up may cause anastomotic leakage despite adequate blood flow before pulling up. We have recently adopted a method of confirming blood flow using ICG fluorescence imaging both before and after pulling up (Fig. 5).

Another important factor is venous return in the gastric wall. As in cases 2 and 3, congestion may cause circulatory disturbance, resulting in mucosal damage, ulceration or poor healing, followed by anastomotic leakage. Current ICG fluorescence imaging evaluates only arterial blood flow at the density or speed of ICG; therefore, a new method is needed for evaluation of the disappearance of ICG reflecting venous return.

After encountering these three cases, we now check carefully for compression by the SCJ at the anastomotic site. Also, the current policy at our institution is as follows: check the shape of the structure at the thoracic inlet on preoperative computed tomography scans, construct a narrow gastric conduit measuring 3.0–3.5 cm in width to reduce compression, secure an inlet space that is as wide as possible by dilating the retrosternal route using Mikulicz’s gauze and evaluate the gastric blood flow by ICG fluorescence imaging before and after pulling up intraoperatively. If there is still a problem at the anastomotic site because of compression by the SCJ, converting the route from retrosternal to subcutaneous or a 2-staged reconstruction procedure might be considered.

## Conclusion

We have encountered three cases in which impaired gastric blood flow behind the SCJ possibly caused anastomotic leakage. When performing reconstruction using the retrosternal route after esophagectomy, it is important to ensure that compression by the SCJ does not adversely affect blood flow at the tip of the gastric conduit.

## Data Availability

All data supporting the findings of this work are available within the article.

## Abbreviations

ICG: Indocyanine green
POD: Postoperative day
SCJ: Sternoclavicular joint
TIS: Thoracic inlet space
STD: Sterno-tracheal distance
VATS: Video-assisted thoracic surgery

## References

[1] Kurahashi Y, Ishida Y, Kumamoto T, Igeta M, Takemura M, Shinohara H (2021). Anastomosis behind the sternoclavicular joint is associated with increased incidence of anastomotic stenosis in retrosternal reconstruction with a gastric conduit after esophagectomy. Dis Esophagus.

[2] Kunisaki C, Makino H, Akiyama H, Nomura M, Otsuka Y, Ono HA (2008). Predictive factors for anastomotic leakage in the neck after retrosternal reconstruction for esophageal cancer. Hepatogastroenterology.

[3] Inoue S, Yoshida T, Nishino T, Goto M, Furukita Y, Yamamoto Y (2020). The sterno-tracheal distance is an important factor of anastomotic leakage of retrosternal gastric tube reconstruction after esophagectomy. Esophagus.

[4] Mine S, Watanabe M, Okamura A, Imamura Y, Kajiyama Y, Sano T (2017). Superior thoracic aperture size is significantly associated with cervical anastomotic leakage after esophagectomy. World J Surg.

[5] Koyanagi K, Ozawa S, Oguma J, Kazuno A, Yamazaki Y, Ninomiya Y (2016). Blood flow speed of the gastric conduit assessed by indocyanine green fluorescence. New predictive evaluation of anastomotic leakage after esophagectomy. Medicine.

[6] Yukaya T, Saeki H, Kasagi Y, Nakashima Y, Ando K, Imamura Y (2015). Indocyanine green fluorescence angiography for quantitative evaluation of gastric tube perfusion in patients undergoing esophagectomy. Am Coll Surg.

